# Application of evoked response audiometry for specifying aberrant gamma oscillations in schizophrenia

**DOI:** 10.1038/s41598-021-04278-5

**Published:** 2022-01-07

**Authors:** Masaya Yanagi, Aki Tsuchiya, Fumiharu Hosomi, Satoshi Ozaki, Osamu Shirakawa

**Affiliations:** 1 Department of Neuropsychiatry, Faculty of Medicine, Kindai University, 377-2 Ohnohigashi, Osaka-sayama, Osaka, 589-8511 Japan; 2 Izumigaoka Hospital, Izumi, Osaka, Japan

**Keywords:** Schizophrenia, Biomarkers

## Abstract

Gamma oscillations probed using auditory steady-state response (ASSR) are promising clinical biomarkers that may give rise to novel therapeutic interventions for schizophrenia. Optimizing clinical settings for these biomarker-driven interventions will require a quick and easy assessment system for gamma oscillations in psychiatry. ASSR has been used in clinical otolaryngology for evoked response audiometry (ERA) in order to judge hearing loss by focusing on the phase-locked response detectability via an automated analysis system. Herein, a standard ERA system with 40- and 46-Hz ASSRs was applied to evaluate the brain pathophysiology of patients with schizophrenia. Both ASSRs in the ERA system showed excellent detectability regarding the phase-locked response in healthy subjects and sharply captured the deficits of the phase-locked response caused by aberrant gamma oscillations in individuals with schizophrenia. These findings demonstrate the capability of the ERA system to specify patients who have aberrant gamma oscillations. The ERA system may have a potential to serve as a real-world clinical medium for upcoming biomarker-driven therapeutics in psychiatry.

## Introduction

Gamma oscillations whose impairments are predominantly shown in the prefrontal cortex are promising clinical biomarkers that may address novel therapeutic interventions for schizophrenia^1–7^. These are neural, rhythmic fluctuations in the gamma frequency range (30–200 Hz) that are commonly captured by electroencephalogram (EEG) or magnetoencephalography^2,8^. Gamma oscillations play a role in information processing in higher-order brain functions (e.g., perception, attention, and working memory). Hence, various types of sensory and cognitive stimuli can elicit gamma oscillations in the associated brain regions^2,6,8^. The auditory steady-state response (ASSR) is a sustained neural entrainment to periodic auditory stimuli, which can probe the ability to generate gamma oscillations by temporally modulating the stimuli at gamma frequency ranges. ASSR potentials in humans are largest when the periodic auditory stimuli are presented at a frequency of approximately 40 Hz^9^.

Previous ASSR studies have shown that the 40-Hz gamma oscillations are highly impaired in patients with schizophrenia^10^. The impaired 40-Hz gamma oscillations implicate abnormal functional interaction between parvalbumin-positive GABAergic neurons and pyramidal neurons in the prefrontal cortex of schizophrenia patients^11–13^. Considering the evidence, provided by rodent studies, that the cortical parvalbumin-positive GABAergic neurons are the generators of gamma oscillations^14,15^, the parvalbumin-positive GABAergic neurons could be the primary target in the treatment of impaired gamma oscillations^16^. Based on the findings of postmortem brain studies, the cortical parvalbumin-positive GABAergic neurons are impaired in the prefrontal cortex of patients with schizophrenia^1,17^, and GABAergic compounds are challenged to compensate for the dysfunction of the GABAergic neurons in schizophrenia patients^18–20^. Kv3.1 is a potassium channel involved in the firing of parvalbumin-positive GABAergic neurons^21^. A compound for modulating Kv3.1 activity has been developed to treat schizophrenia^22^ based on postmortem findings of the brain, the prefrontal reduction of this channel protein in schizophrenia patients^23^. However, given the heterogeneous nature of schizophrenia, the therapeutic targets of such compounds may be optimal for some, but not all, schizophrenia patients^24,25^. Given that gamma oscillations have the potential to detect such targeting pathophysiology among patients with schizophrenia, a simple system to assess the gamma oscillations will be required in the future in psychiatry practice.

ASSR is currently used in clinical otolaryngology for evoked response audiometry (ERA) with medically approved devices. The ASSR in the ERA system is performed by means of an automated analysis that is designed to judge hearing loss by focusing on the phase-locked response detectability. These devices are globally available at many hospital facilities, and the established clinical protocol for measurements ensures the reproducibility of the ASSR results. Furthermore, the 40-Hz ASSR is the testing condition in ERA for awake adults, and this configuration is preset in the devices with a stimulus rate of around 40 Hz. In the standard ERA device, Audera (Grason-Stadler Inc., Eden Prairie, MN, USA), this test is set as an ASSR by the stimulation of a 46-Hz amplitude modulation (AM) tone with slight frequency modulation (FM). That is, the test was set as a 46-Hz AM-FM ASSR. To utilize the plausible availability of the ERA system, this study examined the detectability of impaired gamma oscillations in patients with schizophrenia using the ERA device with the 46-Hz AM-FM ASSR as well as an ASSR with a basic 40-Hz AM tone stimulation, (i.e., 40-Hz AM ASSR).

## Results

### Phase-locked/non-phase-locked response

**Figure 1:**
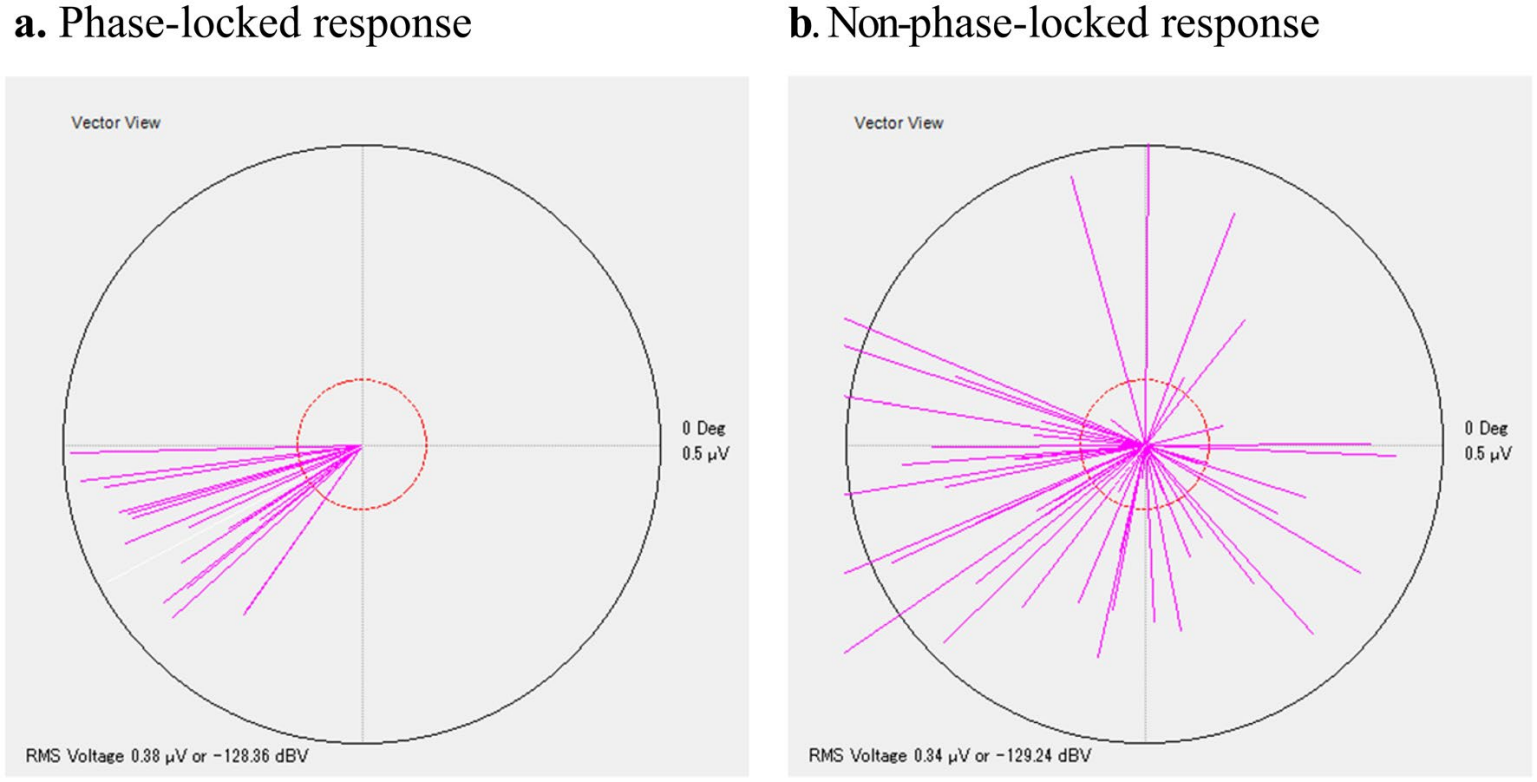
The representative phase-locked (**a**) and non-phase-locked (**b**) responses in 46-Hz AM-FM ASSR in the ERA system. (**a**) and (**b**) are cases of a healthy participant and a patient with schizophrenia, respectively. Each vector in the diagram, which was created by each trial during the auditory stimulus, represents the phase and the amplitude of EEG activity corresponding to the tone modulation frequency rate. The phase angle of the vectors corresponds to the time delay between the presentation of the stimulus and the neural response of trials. The cross-trial phase consistency of the vectors determines the phase-locked or non-phase-locked response. While all trials are aligned in the same range in (**a**), the nonaligned trials that prevent the identification of phase-locked response are seen in (**b**). The mean length of the vectors represents the response amplitude (root mean square voltage, in microvolts) of the ASSR.

Figure 1 shows the phase-locked (A) and non-phase-locked (B) representative responses in 46-Hz AM-FM ASSR. All of the measurement conducted thrice in the healthy subjects (n = 38) demonstrated a phase-locked response in both 46-Hz AM-FM ASSR and 40-Hz AM ASSR. Conversely, a non-phase-locked response was demonstrated in 14 patients in 46-Hz AM-FM ASSR and 10 patients in 40-Hz AM ASSR among the 38 patients with schizophrenia. The details of the occurrence of the phase- and non-phase-locked responses are provided in Table 1, and the results for each patient are in the Supplementary Table. All patients who showed non-phase-locked responses in 40-Hz AM ASSR also showed non-phase-locked responses in 46-Hz AM-FM ASSR, whereas some patients showed non-phase-locked responses only in 46-Hz AM-FM ASSR. The difference between the two ASSRs in terms of the number of patients with non-phase-locked responses was not significant (*P* = 0.46, Fisher’s exact test).

**Table 1:** Occurrence of the phase-locked and non-phase-locked responses in thrice measurements of 40-Hz ASSR.

ASSR		Occurrence of responses for phase-locked/non-phase-locked				Fisher's exact test
		3/0	2/1	1/2	0/3	
46-Hz AM-FM	Healthy subjects, n = 38	38	0	0	0	< 0.0001
	Patients with schizophrenia, n = 38	24	5	5	4	
40-Hz AM	Healthy subjects	38	0	0	0	0.001
	Patients with schizophrenia	28	3	4	3	

### Clinical variables in patients with and without non-phase-locked responses

We defined the all phase-locked group as those who manifested phase-locked responses in all three ASSR measurements and the non-phase-locked group as the patients who manifested at least one non-phase-locked response in the three ASSR measurements. In the logistic regression analysis, significant differences were found between the two groups for both ASSRs in terms of age and in terms of classification of antipsychotics for the 40-Hz AM ASSR (Table 2). No other significant differences were found in terms of other clinical variables between the two groups in either the 46-Hz AM-FM ASSR or the 40-Hz AM ASSR.

**Table 2:** Comparison of clinical variables between all phase-locked and non-phase-locked groups in schizophrenia.

	All phase-locked^a^	Non-phase-locked^b^	*P* value
**46-Hz AM-FM ASSR**			
Number of cases	24	14	
Gender (male/female)	12/12	8/6	0.61
Age, y, mean ± SD	44.0 ± 11.1	51.4 ± 9.1	0.03
Illness duration, y	20.3 ± 11.8	27.1 ± 11.4	0.73
GAF	35.9 ± 15.5	31.1 ± 17.2	0.12
BPRS, four-dimensional model			
Thinking disturbance^c^	5.0 ± 3.3	8.1 ± 4.8	0.20
Withdrawal/retardation^d^	4.5 ± 3.4	5.9 ± 4.9	0.27
Hostile/suspiciousness	2.6 ± 2.9	3.0 ± 2.9	0.06
Anxious/depression	2.7 ± 2.3	3.2 ± 2.4	0.07
Antipsychotics^e^, mg/day	700 ± 99	1388 ± 336	0.06
Classification of antipsychotics^f^	12/12	3/11	0.06
**40-Hz AM ASSR**			
Number of cases	28	10	
Gender (male/female)	16/12	4/6	0.83
Age, y, mean ± SD	45.0 ± 11.3	51.5 ± 8.1	0.05
Illness duration, y	21.3 ± 12.3	27.0 ± 10.7	0.69
GAF	35.0 ± 16.1	31.6 ± 16.4	0.14
BPRS, four-dimensional model			
Thinking disturbance	5.4 ± 3.2	8.3 ± 5.8	0.11
Withdrawal/retardation	4.5 ± 3.3	6.7 ± 5.3	0.10
Hostile/suspiciousness	2.8 ± 3.1	2.6 ± 2.3	0.12
Anxiety/depression	2.9 ± 2.3	2.8 ± 2.3	0.18
Antipsychotics, mg/day	901 ± 183	1099 ± 223	0.75
Classification of antipsychotics	13/15	2/8	0.05

^a^The group of patients who had the phase-locked responses in all three measurements (patients without non-phase-locked responses).

^b^The group of patients who had non-phase-locked responses, at least one in three measurements (patients with non-phase-locked responses).

^c^Positive symptoms.

^d^Negative symptoms.

^e^Chlorpromazine equivalent dose.

^f^New generation antipsychotics only/Conventional antipsychotics or combination (new generation and conventional antipsychotics).

### Number of trials to achieve phase-locked response

**Figure 2:**
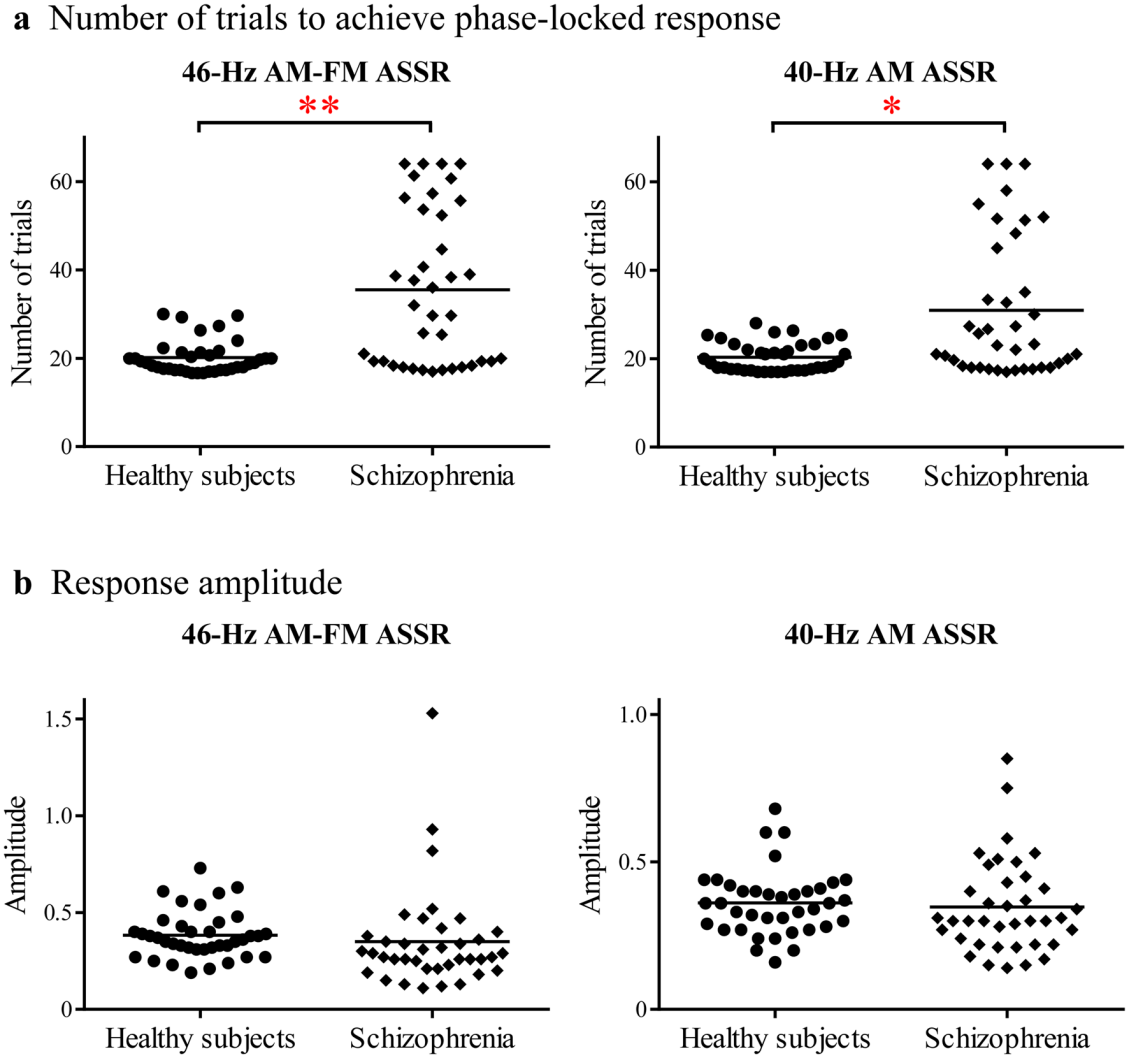
Trends to achieve the phase-locked response and the ASSR potentials. (**a**) Significant differences were observed in the number of trials conducted to achieve a phase-locked response between patients with schizophrenia and healthy subjects in 46-Hz AM-FM ASSR (*left*) and 40-Hz AM ASSR (*right*) . **P < 0.0001, *P = 0.0001. (**b**) No significant differences were observed between patients with schizophrenia and healthy subjects in terms of the response amplitudes of 46-Hz AM-FM ASSR (*left*) and 40-Hz AM ASSR (*right*).

To further investigate the details of ASSR, additional parameters were examined. Significant increases were noted in the number of trials required to achieve a phase-locked response in patients with schizophrenia (46-Hz AM-FM ASSR, mean ± SD = 35.5 ± 17.4; 40-Hz AM ASSR, 31.0 ± 15.9) as compared with healthy subjects (46-Hz AM-FM ASSR, 20.2 ± 3.8; 40-Hz AM ASSR, 20.3 ± 3.3) in either of the ASSRs (46-Hz AM-FM ASSR, *t* = 5.3, *df* = 74, *P* < 0.0001; 40-Hz AM ASSR, *t* = 4.0, *df* = 74, *P* = 0.0001) (Fig. 2a).

### Response amplitude

The response amplitude of the 40-Hz ASSR did not significantly differ between patients with schizophrenia (46-Hz AM-FM ASSR, 0.35 ± 0.26 µV; 40-Hz AM ASSR, 0.35 ± 0.16 µV) and healthy subjects (46-Hz AM-FM ASSR, 0.38 ± 0.12 µV; 40-Hz AM ASSR, 0.36 ± 0.11 µV) in either of the ASSRs (46-Hz AM-FM ASSR, *t* = 0.72, *df* = 74, *P* = 0.47; 40-Hz AM ASSR, *t* = 0.43, *df* = 74, *P* = 0.67). These results are depicted in Fig. 2b. In addition, no significant differences were observed between patients with non-phase-locked responses (46-Hz AM-FM ASSR, 0.34 ± 0.36 µV; 40-Hz AM ASSR, 0.34 ± 0.18 µV) and those without non-phase-locked responses (46-Hz AM-FM ASSR, 0.35 ± 0.19 µV; 40-Hz AM ASSR, 0.35 ± 0.15 µV) in either of the ASSRs (46-Hz AM-FM ASSR, *t* = 0.08, *df* = 36, *P* = 0.94; 40-Hz AM ASSR, *t* = 0.28, *df* = 36, *P* = 0.78).

### Test–retest reliabilities of the ASSR measurements

The number of trials to achieve the phase-locked response and the response amplitude showed consistent results among the three measurements (as shown in Fig. S1) for the entire sample of patients and healthy subjects. Table 3 shows the ICCs among the three ASSR measurements. The average-measure intraclass correlation coefficients (ICCs) showed almost perfect reliabilities for both ASSRs in regard to both the number of trials required to achieve a phase-locked response and the response amplitude (Table 3). The single-measure ICCs showed substantial reliabilities for all conditions except for the response amplitude in 46-Hz AM-FM ASSR, which showed almost perfect reliability (Table 3).

**Table 3:** Test–retest reliabilities by intraclass correlation coefficient (ICC) among thrice measurements of ASSRs.

	Number of trials to achieve the phase-locked response		Response amplitude	
	46-Hz AM-FM ASSR	40-Hz AM ASSR	46-Hz AM-FM ASSR	40-Hz AM ASSR
Average-measure ICC 95% CI	0.92^a^	0.89^a^	0.94^a^	0.92^a^
	0.88–0.95	0.83–0.92	0.92–0.96	0.88–0.94
Single-measure ICC 95% CI	0.79^b^	0.72^b^	0.85^a^	0.78^b^
	0.71–0.85	0.62–0.80	0.79–0.90	0.70–0.85

^a^Almost perfect.

^b^Substantial.

## Discussion

The current study proposes an approach that uses an automated ERA system to specify individual patients with schizophrenia who have severely impaired 40-Hz gamma oscillations. These findings may serve to address the biological variability in schizophrenia patients as it relates to upcoming biomarker-driven therapeutics. The ERA system, which showed excellent detectability of the phase-locked response in healthy subjects, sharply captured the non-phase-locked responses in individuals with schizophrenia in both 46-Hz AM-FM ASSR and 40-Hz AM ASSR (Table 1). Almost perfect test–retest reliabilities were shown among the three measurements of each ASSR in its average-measure ICC for the number of trials required to achieve a phase-locked response, which is the index by which phase-locked response is judged (Table 3). This reproducibility of the repeated ASSR measurements via automatic analysis supports the potential of the ERA system in clinical settings to specify the patients who have severe impairments, which present as non-phase-locked responses in 40-Hz ASSR. Furthermore, the advanced utility of the ERA system in the real-world clinical setting suggests its potential utility in real-world clinical applications in psychiatry. The global ERA system is suitable for use in extensive clinical trials to recruit patients compatible with novel treatments to improve 40-Hz gamma oscillation abnormalities. These biomarker-driven treatments can then be expanded into clinical practices, in which the established clinical procedures of ASSR ensure the reproducibility of results across facilities, globally. Thus, the ERA system could significantly enhance the clinical applications of 40-Hz ASSR due to its simple and globally advanced utility regarding the development of novel and targeted treatments to ameliorate impaired gamma oscillations in schizophrenia patients.

Further clinical investigation into these ASSRs between the all phase-locked and non-phase-locked groups in patients with schizophrenia revealed significant differences in age across both ASSRs as well as in the classification of antipsychotics in the 40-Hz AM ASSR (Table 2). A similar trend was found in the classification of antipsychotics in the 46-Hz AM-FM ASSR (Table 2). Although these findings need to be confirmed in larger cohorts before a conclusion can be drawn, a previous study reported that 40-Hz ASSR was enhanced in patients with new generation antipsychotics as compared to conventional antipsychotics^26^. These findings suggest that the further development of new generation antipsychotics whose effects go beyond that of a dopaminergic blockade may ameliorate the impaired 40-Hz ASSR in patients with schizophrenia.

The results of the current study showed that non-phase-locked responses were observed more frequently in 46-Hz AM-FM ASSR as compared with 40-Hz AM ASSR in schizophrenia patients although the difference between the two ASSRs in terms of these frequencies was not statistically significant. A major difference between the auditory stimuli of 46-Hz AM-FM ASSR and 40-Hz AM ASSR is the fluctuation of the tone induced by the FM. Although other differences in the modulated rate (46 Hz vs. 40 Hz) exist between the two stimuli, a previous study exhibited similar abnormalities in ASSR between the 40- and 45-Hz rates in patients with schizophrenia^27^. Therefore, we speculate that the complex tone stimulation in which the FM component was added to the AM is more sensitive than the AM-only treatment in regard to the neural entrainment dysfunction in schizophrenia patients. FM detection is generally crucial for speech perception, especially under noisy conditions (e.g., the presence of competing voices)^28^, and abnormal speech perception has been reported in patients with schizophrenia^29,30^. The FM variation in the 40-Hz ASSR may be worth exploring in further studies in order to develop an understanding of the abnormal speech perception in schizophrenia patients.

Accumulating evidence has shown that, in addition to the reduced phase-locking, the evoked power that quantifies the phase-locked activity in the 40-Hz ASSR is reduced in patients with schizophrenia^10^. The ERA system does not provide the results to examine the evoked power, though it does provide averaged response amplitude that quantifies both phase-locked and non-phase-locked activities together. In both the 46-Hz AM-FM ASSR and the 40-Hz AM ASSR trials, the response amplitudes did not differ significantly between patients with schizophrenia and healthy subjects (Fig. 2b) despite patients having poor phase-locked responses (Fig. 2a). The typical reason for this phenomenon is represented in Fig. 1. The ASSR with the non-phase-locked response consisted of abundant non-phase-aligned trials that prevented the identification of a phase-locked response (Fig. 1b). These non-phase-aligned activities potently occur at similar amplitude levels to those of normal phase-locked activities (Fig. 1a). Previous studies reported that patients with schizophrenia have aberrant gamma oscillations that are heightened induced (stimulus-induced non-phase-locked) gamma activities that are accompanied by a reduced phase-locking of 40-Hz ASSR^31,32^. These aberrant gamma oscillations may be linked with those shown as the prevailing non-phase-aligned measurements during the ASSR trials in this study.

This is an initial study of the ERA system to examine impaired 40-Hz gamma oscillations in schizophrenia patients. Further development of this assessment system is required to optimize into clinical settings in psychiatry. First, the Audera system defines the non-phase-locked response as cases that did not reach a phase-locked response within 64 trials. This cutoff criterion to define a non-phase-locked response needs to be refined as clinical tests with the ERA system progress in psychiatry. However, the cutoff criterion ought not to be much less than 40 trials so as to avoid including the few impairments of 40-Hz ASSR that can be seen even in the healthy subjects in our study (Fig. 2a). Second, further development of the ERA device software is required. The ERA system provides only single summary results for the frequency domain activity, and it does not store the EEG recordings needed for a detailed analysis of the ASSR. Therefore, we cannot compare the results from the ERA system with commonly used parameters such as the evoked power or the phase-locking factor (PLF). This is a limitation of the current ERA system in regard to a comprehensive understanding of the pathophysiology encircling the impaired 40-Hz ASSR in schizophrenia patients. Third, the influence of the methodological difference between the ERA system and the methods employed in previous ASSR studies in schizophrenia needs to be characterized. The ERA system uses continuous auditory tone stimuli, while previous studies for schizophrenia have used discrete stimuli. A time–frequency decomposition analysis, which is currently the standard analytical method in schizophrenia research^33^, has developed with the discrete tone stimuli. This analysis provides not only basic results, such as PLF and evoked power^33^ but also collateral information such as transient onset/offset responses^34^ and the time course of the response to the discrete tones^35–38^. Nonetheless, the continuous tone stimuli have an advantage that can shorten measurement time, which helps the examinee to avoid falling asleep, a factor confounded with decreasing the amplitude of 40-Hz ASSR^39^. Another ingenuity of the ERA system is the use of short epochs for overlay, which can easily enhance the overlay of epochs up to hundreds of times (up to 640 times in Audera) in a short period. This is favorable for reliably detecting the non-phase-locked response. Further studies are needed to integrate the advantages of the ERA system into schizophrenia research, and such integration might diminish the inconsistency of the findings associated with 40-Hz ASSR in studies on schizophrenia.

In this study, we demonstrated the capability of the ERA system to specify aberrant 40-Hz gamma oscillations in patients with schizophrenia. Given that schizophrenia is a heterogeneous disease whose pathophysiology is shared with other psychiatric diseases, such as bipolar disorder^25,34,40^, the ERA system may be used adjunctively with other biomarkers to biologically classify schizophrenia and related disorders by seeking optimal therapeutic targets with 40-Hz ASSR. Further clinical studies with 40-Hz ASSR via an ERA system are warranted so as to inform the construction of a solid biomarker for the target treatment in the concept of the biological classifications of schizophrenia and related disorders.

## Methods

### Subjects

This study recruited 38 patients with schizophrenia (20 males and 18 females) aged between 24 and 67 years old (mean ± SD = 46.2 ± 11.2) and 38 case-matched healthy subjects (20 males and 18 females) aged between 27 and 68 years (46.7 ± 10.9) from Kindai University Hospital and Izumigaoka Hospital. Each patient was diagnosed based on the DSM-5 criteria^41^. Clinical symptoms and social functioning were assessed using the Brief Psychiatric Rating Scale (BPRS)^42^ and the Global Assessment of Functioning (GAF), respectively. Clinical information was obtained from the clinical psychiatrist in charge of each patient and was based on detailed clinical observations during hospitalization and/or long-term follow-up appointments during outpatient treatment. Diagnoses and clinical assessments were verified by two research psychiatrists who were blind to the ASSR data. None of the patients had a history of auditory disorders, neurological disorders, head trauma, electroconvulsive therapy, or substance/alcohol abuse. However, all patients were administered antipsychotics. Fifteen patients with schizophrenia were using new generation antipsychotics only, and 23 patients with schizophrenia were using conventional antipsychotics or a combination of conventional and new generation antipsychotics. The clinical variables of the patients were as follows: illness duration, 22.8 ± 12.0 years; chlorpromazine-equivalent antipsychotic dose, 945.1 ± 896.5 mg/day; and GAF score, 34.1 ± 16.1. The BPRS item scores were categorized into a four-dimensional model according to a previous report^43^:

1. Thinking disturbance (hallucinatory behavior, unusual thought content, and conceptual disorganization): 6.1 ± 4.2.
2. Withdrawal/retardation (emotional withdrawal, blunted affect, and motor retardation): 5.1 ± 4.0.
3. Hostile/suspiciousness (hostility, suspiciousness, and uncooperativeness): 2.8 ± 2.9.
4. Anxious/depression (anxiety, guilt feelings, and depressive mood): 2.9 ± 2.3.

The subsequent report confirms that Thinking Disturbance and Withdrawal–Retardation reflect positive and negative symptoms, respectively^44^. The healthy subjects had no history of psychiatric, neurological, or auditory disorders. This study was approved by the Ethics Committee of the Kindai University Faculty of Medicine and was carried out in accordance with the ethical principles of the Declaration of Helsinki and its subsequent amendments. A complete description of the study was provided to each study participant, and written informed consent was obtained from all of the participants.

### ASSR measurements

The ASSR was performed with a medical ERA device, Audera, following established technical protocol with minor modifications. The Audera device usually tests one ear at a time with TIP-50 insert phones (Grason-Stader Inc.). In this study, the single tube from the insert phone for the left ear was replaced with a bifurcated tube so as to allow for the binaural presentation of tones. The bifurcated tube was placed so as not to touch the body or the clothing to avoid any interfering noises. The EEG was sampled from an electrode placed on the forehead around the middle point between the Fz and Fpz of the International 10–20 system. The electrodes on the left earlobe and the low forehead around the Fpz served as the reference and the ground electrodes, respectively. The electrode impedances were < 5 kΩ. The 40-Hz ASSR potentials were evoked by two kinds of auditory stimuli with intensity levels at 70 dBHL. The two stimuli used in this study are continuous sine wave tones with a carrier frequency (CF) of 1,000 Hz that have either mixed modulation (100% AM plus 10% FM) at 46 Hz, with which a sinusoidal change of the tone volume between 0 and 70 dB and a fluctuation of the CF tone between 900 and 1,100 Hz occur at the rate of 46 Hz, or 100% AM, with which the tone volume sinusoidally changes between 0 and 70 dB at the rate of 40 Hz. The 46-Hz AM-FM ASSR is the test setting in Audera that is used for awake adults, whereas the 40-Hz AM ASSR is one of the basic settings that has been used, although via discrete tones, in prior ASSR studies on schizophrenia^27,32^. The 46-Hz AM-FM ASSR was slightly modulated from the 40-Hz AM ASSR to increase the ASSR potentials via the addition of frequency modulation^45^. The 46-Hz AM-FM ASSR and the 40-Hz AM ASSR were measured thrice for each subject in a counterbalanced manner. The subjects were instructed to sit and relax on a chair, keep their eyes closed, and remain motionless during ASSR measurements to avoid muscular artifact generation. The subjects were asked to open their eyes during a brief break between the three measurements to avoid falling asleep during any subsequent measurement.

### Data acquisition

The presence or absence of the phase-locked response was automatically determined by a statistical algorithm adopted in the Audera system. Although the details of the algorithm are not available in the public domain, the following steps are implemented in the system. First, after eliminating the initial EEG sampling for approximately 10 s as an acclimation period, the system serially segments the continuous EEG sample during the tone stimulation into an epoch around every 0.1 s. Every ten epochs are then overlaid as trials. Each trial is analyzed using a fast Fourier transform to calculate the amplitude and phase in the frequency domain corresponding to the modulation frequency. These outcomes are reported as vectors (Fig. 1). Moreover, the statistical algorithm applied phase coherence squared (PC^2^) to estimate the phase coherence between the trials and the modulation frequency. The PC^2^ value was statistically evaluated by means of a circular variance to test for a 97% confidence criterion, resulting in phase-locked response detection. The PC^2^ value was updated during the measurement each time a new trial was added, and the system algorithm automatically terminated the stimulation and the data sampling when a phase-locked response was detected or when a phase-locked response could not be reasonably detected within 64 trials. In this study, the latter case was defined as a non-phase-locked response. The minimum number of trials to achieve a phase-locked response is 16, which represents the evaluation of 160 sampling epochs (10 epochs/trial times 16 trials). The maximum number of trials is 64, which resulted in the evaluation of 640 sampling epochs (10 epochs/trial times 64 trials). The time taken to run one measurement ranges from 31 to 98 s, according to the number of trials performed. To support the evaluation of the 40-Hz gamma oscillations in the presence or absence of a phase-locked response, additional parameters were selected: (1) the number of trials to achieve a phase-locked response, which was displayed as the number of vectors in Fig. 1, and (2) the response amplitude (root mean square voltage; in microvolts) of the ASSR, which was calculated as the mean length of the vectors. The non-phase-locked response, which was judged not to phase-lock within 64 trials, was assigned the maximum trial number of 64 in the statistical analysis of the number of trials required to achieve a phase-locked response. The results of three measurements for each parameter were averaged for each of the stimuli for each subject.

### Statistics

The occurrence of the non-phase-locked response was compared between patients with schizophrenia and healthy subjects using Fisher’s exact test. The clinical variables were compared between the all phase-locked and non-phase-locked groups of patients using binary logistic regression. The numbers of trials required to achieve the phase-locked response and the response amplitude were compared between the two groups via unpaired *t*-tests. The test–retest reliabilities for the ASSR measurements were examined across the entire sample of patients and healthy subjects using ICCs^46,47^ in terms of the number of trials required to achieve the phase-locked response and the response amplitude. A one-way random, single-measure ICC analysis and a one-way random, average-measure ICC analysis were performed to examine the reliabilities of single and triple measurements of ASSR, respectively. According to a previous report^48^, the quality of ICC was judged as follows; ICC < 0.00, poor; 0.00 to 0.20, slight; 0.21 to 0.40, fair; 0.41 to 0.60, moderate; 0.61 to 0.80, substantial; 0.81–1.00 almost perfect. All statistical tests were two-tailed, and the threshold for the significance of *P* values was set at 0.05. SPSS version 25.0 (IBM Inc., Armonk, NY, USA) was used for the statistical analyses.

## Supplementary Information

Supplementary Information.

## Data Availability

The data supporting the findings of this study are available from the corresponding author upon reasonable request.
