# Supplementary material for: Application of evoked response audiometry for specifying aberrant gamma oscillations in schizophrenia
Source: Sci Rep. 2022 Jan 7;12:287. doi: 10.1038/s41598-021-04278-5 (PMC8741931; doi:10.1038/s41598-021-04278-5)
Supplement: Supplementary file 1 — Supplementary Information. [file 41598_2021_4278_MOESM1_ESM.docx]

**Supplementary Information**

**Application of Evoked Response Audiometry for Specifying Aberrant Gamma Oscillations in Schizophrenia**

Masaya Yanagi, M.D., Ph.D.^1*^, Aki Tsuchiya, M.D.^1^, Fumiharu Hosomi, M.D., Ph.D.^1^, Satoshi Ozaki, M.D., Ph.D.^2^, and Osamu Shirakawa, M.D., Ph.D.^1^

^1^Department of Neuropsychiatry, Kindai University Faculty of Medicine, Osaka-sayama, Osaka, Japan

^2^Izumigaoka Hospital, Izumi, Osaka, Japan

**Supplementary Table.**

Occurrence of non-phase-locked responses in each patient with schizophrenia

**Supplementary Figure.**


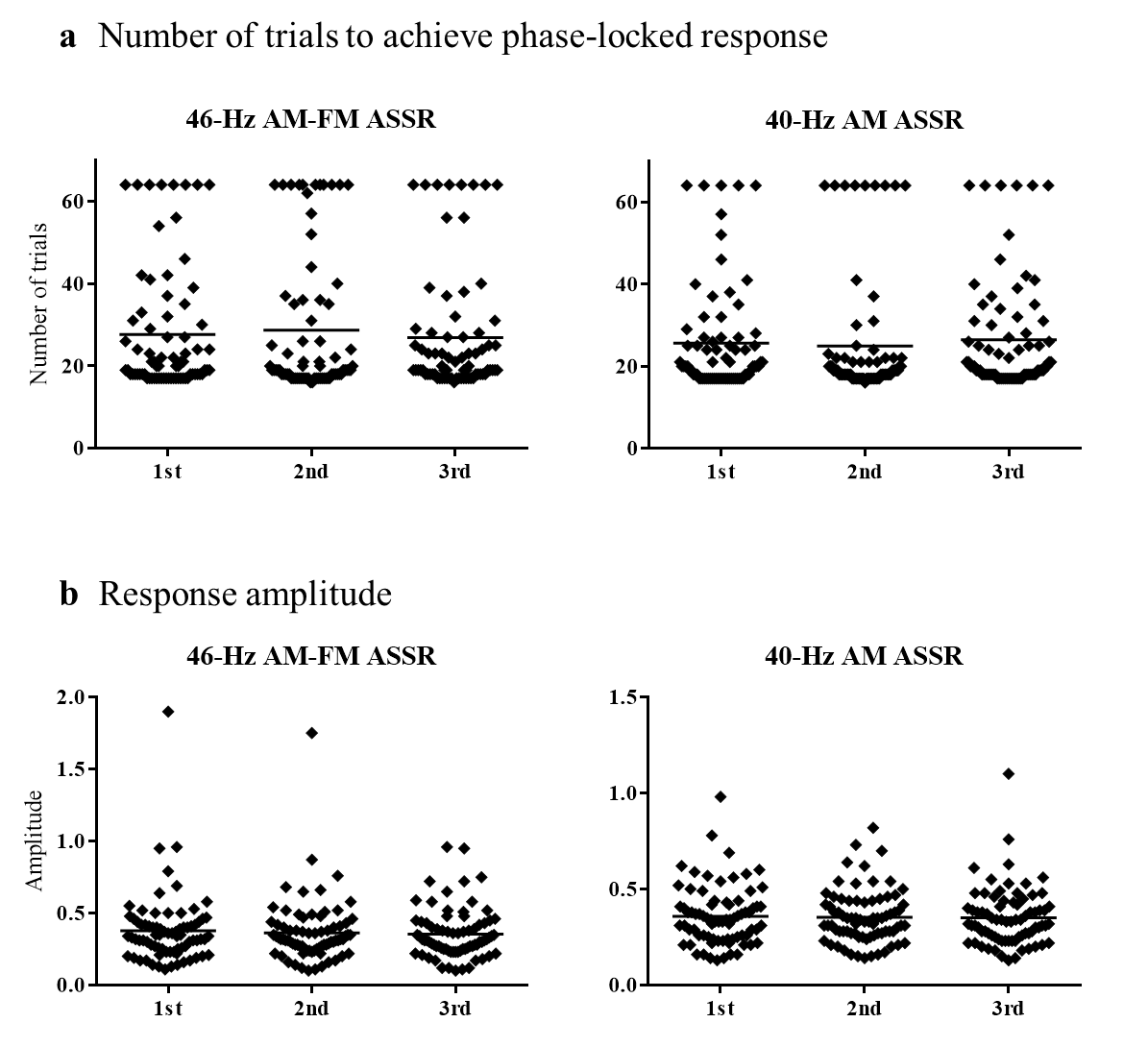


**Figure S1**. Comparison of the number of trials required to achieve a phase-locked response (**a**) and its response amplitude (**b**) across three measurements of ASSRs. No significant differences were observed among these parameters across the different ASSRs (as determined by a one-way ANOVA).

The number of trials required to achieve phase-locked response: *F*(2, 225) = 0.26, *P* = 0.77 for the 46-Hz AM-FM ASSR, and *F*(2, 225) = 0.21, *P* = 0.81 for the 40-Hz AM ASSR.

Response amplitudes: *F*(2,225) = 0.24, *P* = 0.78 for the 46-Hz AM-FM ASSR, and *F*(2,225) = 0.06, *P* = 0.94 for the 40-Hz AM ASSR.
